# Mechanical Behavior of Dowel-Type Joints Made of Wood Scrimber Composite

**DOI:** 10.3390/ma9070581

**Published:** 2016-07-15

**Authors:** Minjuan He, Duo Tao, Zheng Li, Maolin Li

**Affiliations:** 1 Department of Structural Engineering, Tongji University, Shanghai 200092, China; hemj@tongji.edu.cn (M.H.); 91taoduo@tongji.edu.cn (D.T.); 2 Tianjin Hualin (Group) Co., Ltd., 88 Nanhuan Road, Tianjin 300350, China; benilitj@gmail.com

**Keywords:** scrimber composite, bolted joints, mechanical behavior, finite element model, failure mechanism

## Abstract

As a renewable building material with low embodied energy characteristics, wood has gained more and more attention in the green and sustainable building industry. In terms of material resource and physical properties, scrimber composite not only makes full use of fast-growing wood species, but also has better mechanical performance and less inherent variability than natural wood material. In this study, the mechanical behavior of bolted beam-to-column joints built with a kind of scrimber composite was investigated both experimentally and numerically. Two groups of specimens were tested under monotonic and low frequency cyclic loading protocols. The experimental results showed that the bolted joints built with scrimber composite performed well in initial stiffness, ductility, and energy dissipation. A three-dimensional (3D) non-linear finite element model (FEM) for the bolted beam-to-column joints was then developed and validated by experimental results. The validated model was further used to investigate the failure mechanism of the bolted joints through stress analysis. This study can contribute to the application of the proposed scrimber composite in structural engineering, and the developed FEM can serve as a useful tool to evaluate the mechanical behavior of such bolted beam-to-column joints with different configurations in future research.

## 1. Introduction

With low embodied energy and carbon storage characteristics, wood has gained more and more attention in the green and sustainable building industry in recent years. However, wood normally has larger variabilities in mechanical properties compared to concrete or steel. Therefore, different kinds of engineered wood-based composite materials have been developed. Compared to solid sawn lumber, the variability in mechanical properties of wood-based composite is significantly reduced since natural defects in wood are dispersed. The decrease in non-uniformity leads to efficient utilization of fiber resources, and better mechanical performance can be obtained from engineered wood-based composites.

In the past two decades, researches on the structural application of wood-based composites have been conducted. The performance of glulam timber columns reinforced by FRP (Fiber Reinforced Plastic) sheets were investigated by Taheri et al. [1], and experimental results and computational modelling showed that the stiffness and strength could be significantly improved with FRP that were externally bonded. O’Loinsigh et al. [2] conducted experimental and numerical investigations on multi-layered wooden beams with welded-through wood dowels, showing that a desirable bending stiffness is attainable with a reasonable combination of material and geometric parameters. Quiroga et al. [3] investigated the feasibility of rapid housing construction with wood-cement composites. It was shown that such composites are able to compete with existing building materials in structural durability and production cost. In addition, a large amount of researches on the mechanical properties of innovative wood-based composite have been conducted [4,5,6,7,8,9,10,11,12,13,14]. These researches showed that wood-based composites could meet both structural and non-structural demands with desirable mechanical performance and better use of wood resources.

The authors have proposed a kind of veneer-based scrimber composite made from fast-growing wood. The desirable appearance makes such scrimber composite a potential material choice for post-and-beam timber constructions. The mechanical behavior of beam-to-column joints has a significant influence on the seismic performance of a post-and-beam structural system. In this study, the mechanical behavior and failure mode of bolted beam-to-column joints built with the proposed scrimber composite were investigated by monotonic and low frequency cyclic tests. A 3D non-linear finite element model (FEM) was then developed and validated by experimental results, with which the mechanical behavior and failure mechanism of the bolted beam-to-column joints was further investigated. Moreover, to evaluate the feasibility of using the scrimber composite in post-and-beam buildings, the rotational performance of the scrimber joints was compared with that of glulam joints, since glulam is considered as the most commonly used material for existing post-and-beam buildings. The presented experimental and numerical analyses can serve as a fundamental basis for promoting more application of such scrimber composite material in modern timber structures.

## 2. Rotational Performance of Scrimber Beam-to-Column Joints

**Figure 1:**
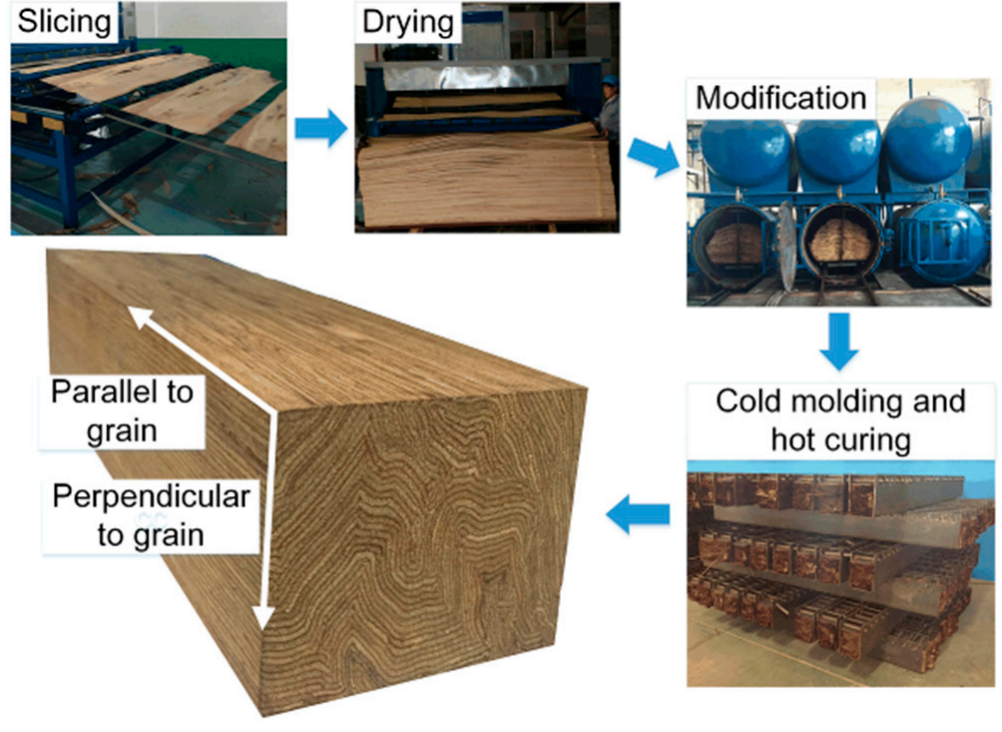
Production and cross section of the scrimber composite.

The production process and typical cross section of the proposed scrimber composite is shown in Figure 1. The logs are firstly sliced into veneer lumber with a thickness of only 2 mm. Then the adhesive impregnation process is conducted on the dried veneer lumber, after which the veneers are squeezed and molded under high temperature and pressure. The cross section of the scrimber composite is characterized by a curved veneer, and this is quite different from the existing wood-based composites that are engineered. The mechanical properties of such scrimber composite have been investigated by Li et al. [15]. With a desirable strength and modulus of elasticity (MOE), it is recommended that the scrimber composite can be used for structural beams and columns for a post-and-beam timber system. In this study, both monotonic and low frequency cyclic tests were conducted to investigate the rotational performance of scrimber beam-to-column joints.

### 2.1. Experimental Description

#### 2.1.1. Specimens

**Figure 2:**
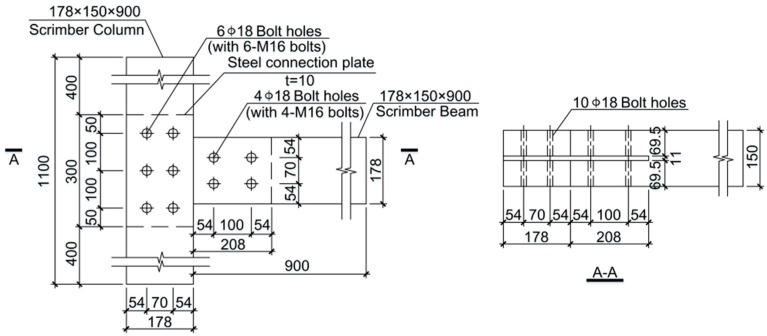
Specimen configuration (all dimensions are in mm).

The configuration of the specimen is shown in Figure 2. The beam member was 178 mm × 150 mm in cross section and 900 mm in length, and the column member had the same cross section as the beam member, but it was 1100 mm in length. A slot with a thickness of 11 mm was cut for the 10 mm-thick steel connection plate, and bolt holes with diameter of 18 mm were pre-drilled for the M16 bolts (Ningbo Xinderui Standard Parts Co., Ltd., Ningbo, China) (i.e., bolts with diameter of 16 mm). Q235B steel plate (manufactured by Qingdao East Steel Tower Stock Co., Ltd., Qindao, China) with a nominal yielding strength of 235 MPa, as specified in the Chinese code of notations for designations of iron and steel [16], was used for the connection plate. Bolts with the grade of 6.8, conforming to Chinese Standard GB/T 1231-2006 [17] were used as fasteners for the specimens. The nominal yielding strength of the bolts was 480 MPa. A total of 10 specimens were prepared for the tests. One group with five duplicates was tested under monotonic loading, and the other group with five duplicates was tested under low frequency cyclic loading.

#### 2.1.2. Test Design and Data Measurement

**Figure 3:**
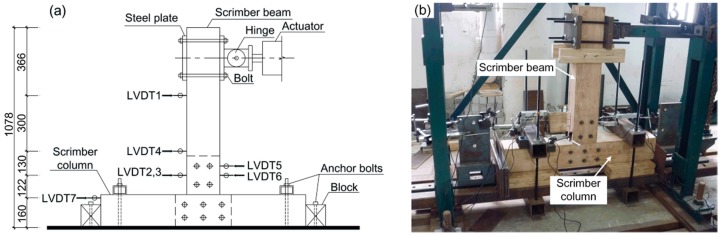
Test design: (**a**) test layout (all dimensions are in mm); (**b**) test set-up in the laboratory.

The specimens were rotated 90 degrees for ease of loading in the laboratory. As shown in Figure 3a, the column was horizontally fixed on the ground by anchor bolts, and the beam was placed vertically with its top end connected to the electro-hydraulic servo actuator. Figure 3b shows the test set-up of the specimen in the laboratory. The actuator has a maximal loading capacity of 300 kN and a stroke range of ±250 mm.

The rotation of the joint was measured by seven linear voltage displacement transducers (LVDTs) (manufactured by Liyang City Instrument and Meter Plant, Liyang, China), which were distributed along the height of specimen as illustrated in Figure 3a. LVDT 1 was used to record the horizontal displacement of the beam’s free end. LVDT 2, LVDT 3, and LVDT 4 were used to measure the beam rotation. LVDT 2 and LVDT 3 were installed on both sides of the steel connection plate symmetrically. LVDT 5 and LVDT 6 were connected to the steel connection plate with a distance of 50 mm to measure the rotation of the steel connection plate. LVDT 7 was installed at the end of the column to record the horizontal rigid body movement of the entire joint.

Considering the centroid of the group of bolts as the rotation center of the joint, the moment and corresponding rotation of the joint can be calculated by Equations (1)–(4).

**Figure 4:**
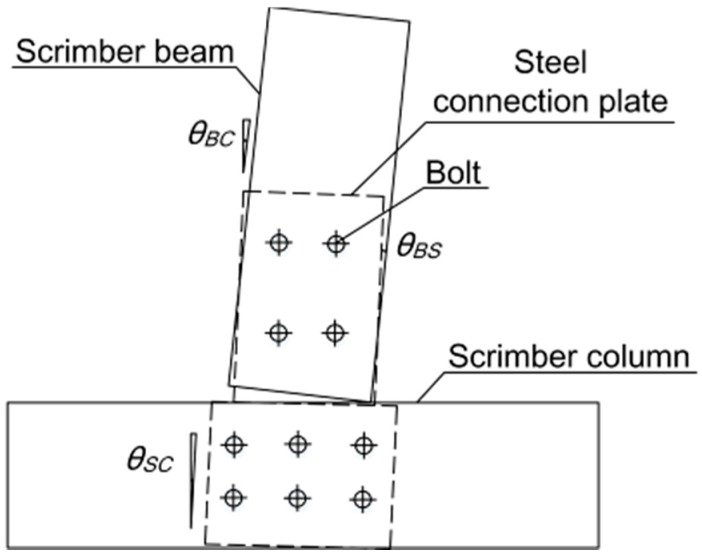
Calculation of relative rotation.

(1) M=F×H

(2) $$\theta =\theta_{BS}=\theta_{BC}-\theta_{SC}$$

(3) $$\theta_{BC}=\left[\text{arctan}\frac{S_{4}-\left(S_{2}{+\text{S}}_{3}\right)/2}{120}\right]\times \frac{180}{\pi }$$

(4) $$\theta_{SC}=\left[\text{arctan}\frac{S_{5}-S_{6}}{50}\right]\times \frac{180}{\pi }$$

where *F* is the lateral force applied on the specimen by the actuator, and *H* is the vertical distance between the rotation center and the loading point. As shown in Figure 4, *θ* is the rotation of the beam relative to the rotation center, *θ_BS_* is the rotation of the beam relative to the steel connection plate, *θ_BC_* is the rotation of the beam relative to the column, and *θ_SC_* is the rotation of the column relative to the steel connection plate. *S2*, *S3*, *S4*, *S5*, and *S6* are the displacements measured by LVDT 2–LVDT 6, respectively.

#### 2.1.3. Loading Protocol

**Figure 5:**
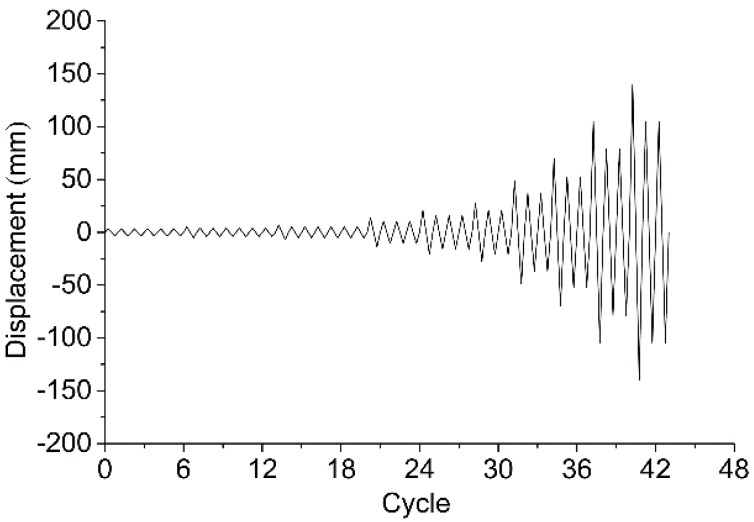
Consortium of Universities for Research in Earthquake Engineering (CUREE) protocol for the low frequency cyclic tests.

Displacement-control protocol was adopted for both monotonic and low frequency cyclic tests. In accordance with American standard ASTM D1761-12 [18], the monotonic loading was applied at a constant rate of 5 mm/min until the failure of specimen occurred (i.e., the loading dropped by more than 20% of the peak value). For the low frequency cyclic tests, the Consortium of Universities for Research in Earthquake Engineering (CUREE) protocol was adopted according to American standard ASTM E2126-11 [19]. As shown in Figure 5, the reference displacement of the CUREE protocol (i.e., Δ) was determined as 60% of the maximal displacement obtained by monotonic tests. The CUREE protocol includes initiation cycles, primary cycles, and trailing cycles. The loading protocol begins with six initiation cycles with an amplitude of 0.05Δ, followed by the first primary cycle with an amplitude of 0.075Δ, and then a rise up to the final primary cycle with an amplitude of 2.0Δ. Each primary cycle is followed by several trailing cycles with 75% of the amplitude of the primary cycle.

### 2.2. Failure Modes

**Figure 6:**
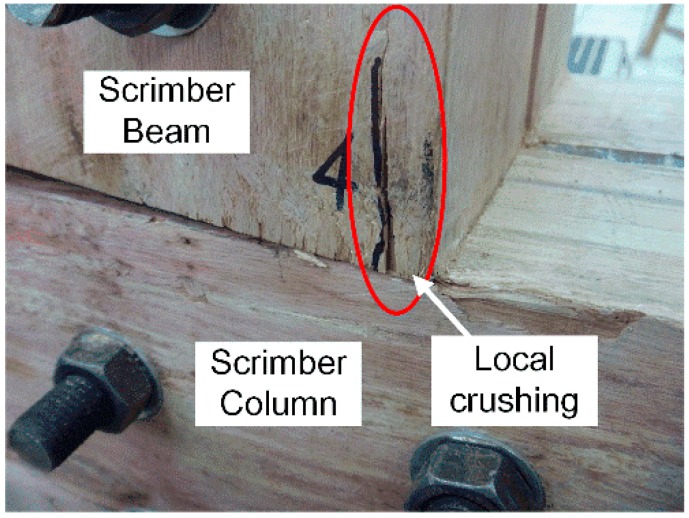
Local crushing between beam and column.

In the beginning stage of loading, the rotation of joints was almost free due to the initial clearance between bolts and wood components. Soon a stop of the potential rotation was observed with partial contact between beam and column on the compression side. The moment-resisting capacity of joints was mainly dependent on the extrusion between beam and column before the clearance was eliminated. With the increase of moment, the local crushing failure appeared with slight crack development on the beam as shown in Figure 6, and then the moment-resisting capacity of joints was mainly dependent on the interaction between steel components and wood components. It is suggested the progress of the rotation of joints could be influenced by the local crushing between elements.

**Figure 7:**
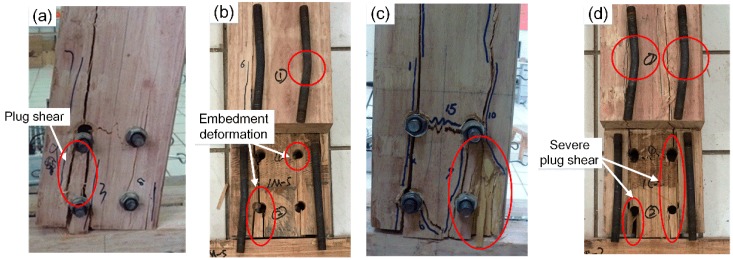
Failure modes: (**a**) external observation from monotonic test; (**b**) internal observation from monotonic test; (**c**) external observation from cyclic test; (**d**) internal observation from cyclic test.

The failure modes of the bolted scrimber joints from monotonic tests are shown in Figure 7a,b. With the increase of rotations, splitting always appeared towards the bottom of beam on the tension side and then developed upwards. At larger rotations, the joint eventually failed with the appearance of run-through crack along the beam, and severe plug shear underneath the bolts was also observed. After disassembly and examination of the failed specimens, bolt yielding was observed with embedment deformation of bolt holes. As shown in Figure 7c,d, the failure modes from cyclic tests were similar to, but more severe than, those from monotonic tests. This was due to the fact that the cyclic loading in positive and negative directions added to the damage accumulation of the specimens, and the maximal displacement of the actuator from cyclic tests (i.e., 2.0Δ) was larger than that from monotonic tests (i.e., about 1.7Δ).

### 2.3. Results of Monotonic Tests

**Figure 8:**
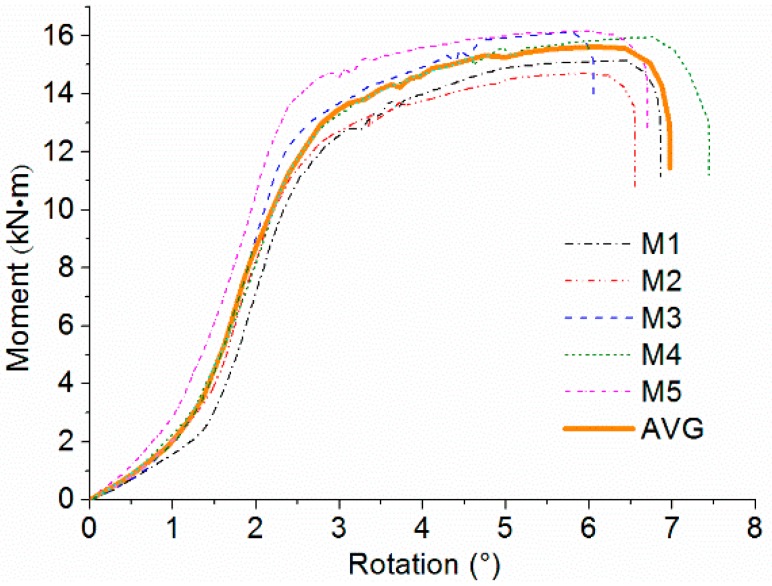
Moment-rotation (*M-θ*) curves and the average *M-θ* curve of monotonic tests.

Figure 8 shows the moment-rotation (*M-θ*) curves from monotonic tests. The first branch of the curve is close to the *x* axis, indicating low moment-resisting capacity of joints caused by initial clearance between bolts and wood components. When the rotation of beam relative to column increased to almost 1.5 degrees, the stiffness of joints significantly increased with sufficient contact between bolts and wood components. It is noted that the five *M-θ* curves are similar with a variation coefficient less than 10%, thus the results can be represented by the average curve as shown in the figure. The average curve is almost linear with the joints in the elastic deformation stage. Due to the load distribution after plug shear failure, a short period of volatility is observed with unstable moment-resisting behavior. Afterwards, there is a yielding plateau in the curve with yielding of bolts, showing that the scrimber joints perform well in ductility.

Glulam is normally used in timber constructions. In order to evaluate the feasibility of using scrimber joints in structural engineering, comparison between the scrimber joints and glulam joints was also performed in this study. Based on the same design target, including stiffness and moment resisting capacity, the dimension of scrimber joints (i.e., the cross sections of scrimber beam and column were both 178 mm × 150 mm) was eventually smaller than that of glulam joints (i.e., the cross sections of glulam beam and column were 300 mm × 200 mm and 300 mm × 250 mm, respectively). The experimental results of the glulam joints were reported by He and Liu [20]. For comparison, the CEN (European Committee for Standardisation) method was adopted to calculate the initial stiffness and ductility ratio [21]. The initial stiffness is defined by the slope of a secant line from 10% to 40% of the peak moment. The yield point is determined as the intersection of the secant line and a tangent line with a slope equal to one sixth of the slope of the secant line. The ductility ratio is determined as the ratio of the maximal rotation to the yield rotation corresponding to the yield point. These key points, used to calculate various performance parameters of joints, are not always located in the first branch of the curve, so the adverse impact on analysis results caused by erection error and initial clearance is effectively eliminated. The comparisons of initial stiffness, ultimate moment, failure rotation, and ductility ratio are given in Table 1. Except the aforementioned design performance, it is noted that the ductility ratio of scrimber joints is 2.28. This is mainly due to the fact that improvements of the material hardness and tensile strength perpendicular to the grain of the scrimber composite enhance the interaction between wood and steel members, leading to yielding of bolts and the delay of cracking. However, the failure mode of glulam joints was brittle, since wood splitting appeared almost simultaneously with the yielding of bolts.

### 2.4. Results of Cyclic Tests

#### 2.4.1. Hysteretic Loop and Backbone Curve

**Figure 9:**
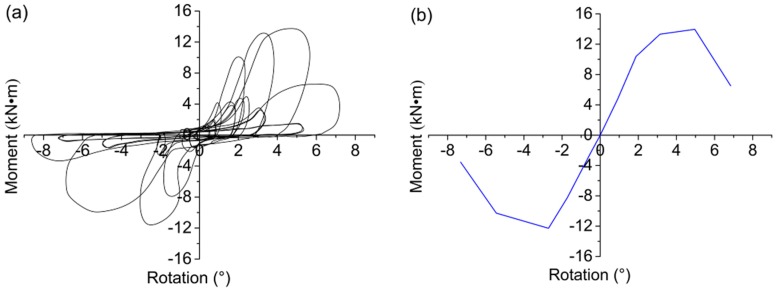
Analyses of cyclic tests: (**a**) typical hysteresis loop; (**b**) backbone curve.

The typical hysteretic loop and corresponding backbone curve from cyclic tests are shown in Figure 9. The typical hysteretic loop shows a reverse “S” shape with significant pinching phenomenon, which is mainly caused by the unrecoverable plastic deformation of wood components. With the increase of rotation, the pinching phenomenon becomes more severe due to the damage accumulation in the specimens. The backbone curve is the envelope obtained by connecting the peak point in each primary cycle of the hysteretic loop. It is noted that the backbone curve is nonlinear with stiffness degradation caused by wood fracture and bolt bending. The backbone curve enters a softening stage in the last primary cycle, showing a decrease of moment-resisting capacity with severe damage of the specimen.

#### 2.4.2. Stiffness Degradation

The stiffness of the joint, which is crucial for the lateral deformability of a post-and-beam timber system, would decline due to the unrecoverable damage such as fracture and embedment deformation of wood components. To evaluate the degree of damage accumulation, the secant stiffness of each primary cycle is calculated by:

(5) $$K_{i}=\frac{\left|+M_{i}\right|+\left|-M_{i}\right|}{\left|+\theta_{i}\right|+\left|-\theta_{i}\right|}$$

where +*M_i_* and −*M_i_* are the positive and negative peak moments of the *i*-th primary cycle, respectively; +*θ_i_* and −*θ_i_* are the rotations corresponding to +*M_i_* and −*M_i_*, respectively.

**Figure 10:**
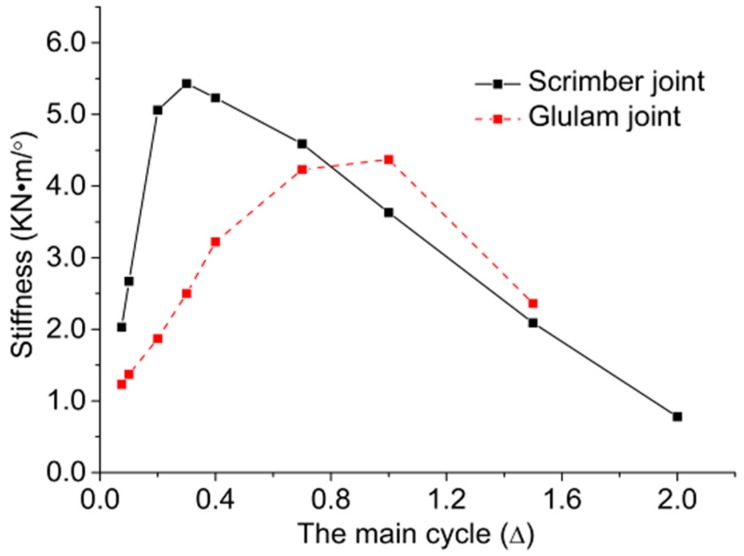
Comparison of average stiffness degradation between scrimber joint and glulam.

The comparison of stiffness degradation between scrimber joints and glulam joints is shown in Figure 10. It is suggested that the secant stiffness of scrimber joints is larger at loading stages from 0.075Δ to 0.7Δ, and the stiffness of glulam joints decreased faster than the stiffness of scrimber joints. This is due to the fact that the failure of scrimber joints was mainly caused by gradual damage accumulation, while the failure of glulam joints was caused by the sudden appearance of run-through crack in the wood. Moreover, the maximal secant stiffness of scrimber joints is about 30% larger than that of glulam joints.

#### 2.4.3. Energy Dissipation

The energy dissipation capacity of different joints can be compared by the equivalent viscous damping ratio (EVDR), which is calculated by:

(6) $$h_{e}=\frac{1}{2\pi }\times \frac{E_{d}}{E_{p}}$$

**Figure 11:**
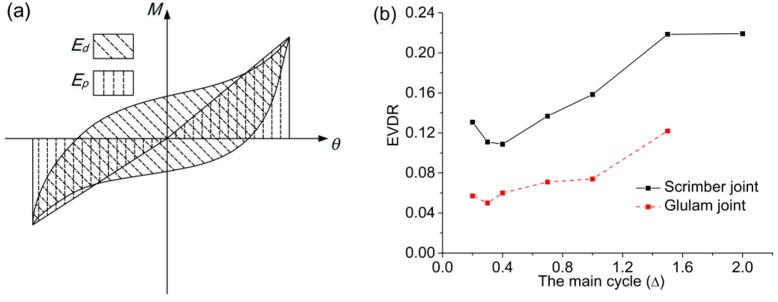
Calculation of equivalent viscous damping ratio (EVDR): (**a**) mathematical meaning of *E_d_* and *E_p_*; (**b**) comparison between scrimber joint and glulam joint.

As illustrated in Figure 11a, *E_d_* is the energy dissipated in one cycle equal to the enclosed area of the hysteretic loop, and *E_p_* is the nominal elastic potential energy equal to the sum of the product of the peak moment and the corresponding rotation in the positive direction and the negative direction.

The comparison of EVDR from 0.2Δ to 2.0Δ is shown in Figure 11b. The EDVR of scrimber joints is about twice that of glulam joints at each primary cycle, showing that the scrimber joints perform well in energy dissipation. With bending deformation of bolts, the EDVR of scrimber joints increases rapidly from 0.4Δ to 1.6Δ. Since bolt yielding has become the major energy dissipation mechanism at the last primary cycle, the EDVR of scrimber joints almost remains unchanged.

## 3. Numerical Analysis

Numerical modelling is an effective way of investigating the mechanical behavior of bolted joints. During the past decade, some numerical research for bolted joints based on two-dimensional (2D) FEM was conducted [22,23]. It was suggested that 2D FEM was only appropriate for some specific situations such as for very thin or very thick wood components. Therefore, a 3D FEM considering the anisotropic elastoplastic behavior of scrimber composite was developed and validated by experimental results. The validated model was further used to investigate the mechanical behavior and failure mechanism of bolted scrimber joints through stress analyses combined with parametric analyses.

### 3.1. Finite Element Model

#### 3.1.1. Material Properties

The steel is regarded was isotropic with Young’s modulus equal to 210 GPa and Poisson’s ratio equal to 0.3. The stress-strain properties of bolts and steel plate were both assumed as a bi-linear hardening model. The yielding strength and ultimate strength of steel connection plate were 270 MPa and 550 MPa, respectively, with ultimate strain equal to 0.2. The yielding strength and ultimate strength of bolts were 480 MPa and 600 MPa, respectively, with ultimate strain equal to 0.1. All the stress-strain parameters were determined by tension tests.

The scrimber composite was modelled as transverse isotropy material with identical characteristics in radial and tangential directions. For bolted timber joints, the bearing capacity was mainly governed by the embedment properties of wood and bending resistance of bolts. Hong et al. [24,25] found the MOE obtained by embedment tests was quite different from that obtained by compression tests. Therefore, a so-called embedment region around the bolt holes was defined with a cross section of 2.5 d. × 2.5 d. (d. is the diameter of bolts). The MOE of the embedment region was taken by reducing the value from material tests. The reduction factor was taken as 0.2 in the parallel-to-grain direction and 0.7 in the perpendicular-to-grain direction based on previous experience.

As the generalized version of Von-Mises yield criterion, the Hill criterion was adopted to consider the orthotropy of the scrimber composite [26]. The equivalent stress can be expressed by Equation (7):

(7) $$\bar{\sigma }={\left[a_{1}{\left(\sigma_{y}-\sigma_{z}\right)}^{2}+a_{2}{\left(\sigma_{z}-\sigma_{x}\right)}^{2}+a_{3}{\left(\sigma_{x}-\sigma_{y}\right)}^{2}+3a_{4}\tau_{zx}^{2}+3a_{5}\tau_{yz}^{2}+3a_{6}\tau_{xy}^{2}\right]}^{1/2}/\sqrt{2}$$

where $a_{1}=\frac{2}{f_{c,90}^{2}}-\frac{1}{f_{c,0}^{2}},\;a_{2}=a_{3}=\frac{1}{f_{c,0}^{2}},\;a_{4}=a_{5}=a_{6}=\frac{2}{3f_{v}^{2}}$; *σ_i_* and *τ_ij_* are the normal stress and shear stress of the scrimber composite, respectively; *f_c,_*_0_ and *f_c,_*_90_ are the compressive strengths parallel and perpendicular to grain, and *f_v_* is the shear strength of the scrimber composite.

**Figure 12:**
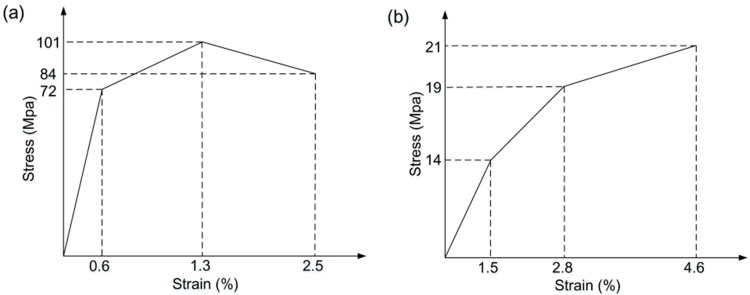
Stress-strain properties of scrimber composite: (**a**) parallel-to-grain; (**b**) perpendicular-to-grain.

The stress-strain properties of the scrimber composite in parallel-to-grain and perpendicular-to-grain directions are defined by two different multi-linear kinematic hardening models (i.e., KINH models in ANSYS) as shown in Figure 12, which are combined with the Hill criterion to simulate the elastic-plastic behavior of the material [27]. Based on the material tests, the mechanical properties of the scrimber composite used in the model are given in Table 2.

#### 3.1.2. Crack Simulation

**Figure 13:**
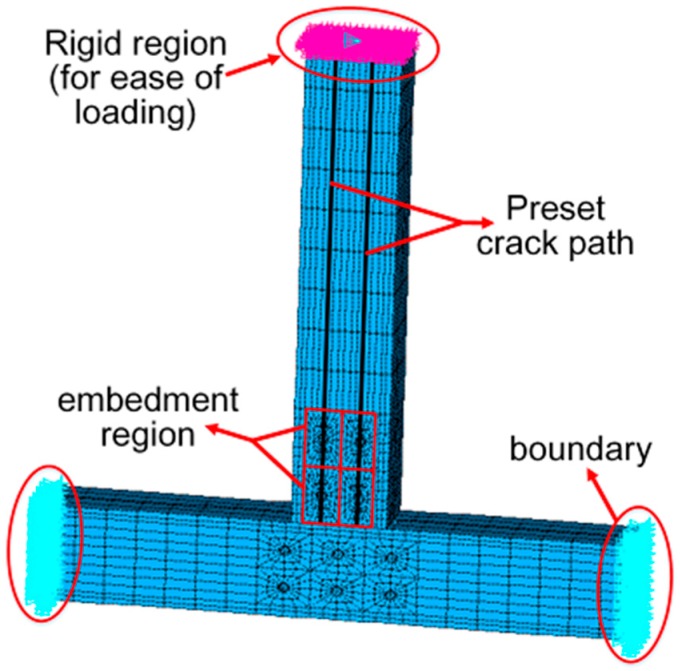
The 3D finite element model (FEM).

To simulate the failure process of bolted joints explicitly, the cohesive zone material (CZM) was used to model the crack [28]. Contact elements were set on both sides of the designed crack path as shown in Figure 13. The element pairs would be separated according to the stress distribution of the defined CZM, and then a new crack surface was created. The available CZM in the software ANSYS is based on the models proposed by Alfano and Crisfield [29], which includes Mode I, Mode II, and the mixed mode. Considering the combined effect of perpendicular-to-grain tensile stress and parallel-to-grain shear stress, the mixed model is chosen to realize the fracture criteria as illustrated in Equation (8):

(8) $$\frac{G^{I}}{G_{c}^{I}}+\frac{G^{II}}{G_{c}^{II}}=1$$

where $G^{I}$ and $G_{c}^{I}$ are the fracture energy and the critical fracture energy considering normal contact stress; $G^{II}$ and $G_{c}^{II}$ are the fracture energy and the critical fracture energy considering tangential contact stress, respectively. Since it is difficult to determine the values of $G_{c}^{I}$ and $G_{c}^{II}$ through material tests, in this study, $G_{c}^{I}$ and $G_{c}^{II}$ were calibrated versus the joint test results. As shown in Table 2, the critical fracture energy $G_{c}^{I}$ and $G_{c}^{II}$ are taken as 0.56 N/mm and 1.32 N/mm, respectively.

#### 3.1.3. Contact Simulation

Contacts exist in the interface between: the scrimber members and steel plate, the bolts and scrimber members, the bolts and steel plate, and the scrimber beam and column members. The interaction between different components was simulated by surface-to-surface contact pairs, where the surface with higher rigidity was defined as the target surface, and the surface with lower rigidity was defined as the contact surface. The normal pressure and tangential friction force were transferred between the contact pairs without considering penetration. The initial normal stiffness was dependent on the material characteristics of the softer surface. The friction was evaluated by the Coulomb stick-skip criterion, which allows no relative movement until the friction force is reached. According to the research on bolted timber joints [30], the friction coefficient between scrimber and bolt, and scrimber and steel connection plate were set as 0.6 and 0.1, respectively. The friction coefficient between bolt and steel connection plate, and scrimber beam and column were taken as 0.2 and 0.5, respectively.

#### 3.1.4. Meshing and Boundary Conditions

The 8-node hexahedral elements with reduced integration, which exists in ANSYS element library, were used to model steel and wood components. Each node of the elements has three translational degrees of freedom in directions of *x*, *y,* and *z*, respectively. To track the whole deformation process of the joints, the Newton-Raphson method was chosen as the incremental scheme. Considering the high MOE of scrimber composite, the reduced integration could prevent nearly incompressible elements from volume locking. However, the artificial strain energy, which was introduced to control the energy spurious mode, may have an effect on the calculation accuracy. With the ratio of artificial strain energy to the total strain energy being less than 5%, the calculation accuracy was almost within the limit of error. On the contrary, the elements should be remeshed to increase the number of elements and the mesh density of some irregular areas. Moreover, some other techniques in modeling could be adopted to effectively solve the problems appeared in meshing [31,32,33]. Two types of boundary conditions were considered, including those due to the loading and those due to the displacements as shown in Figure 13.

### 3.2. Validation of the Numerical Model

**Figure 14:**
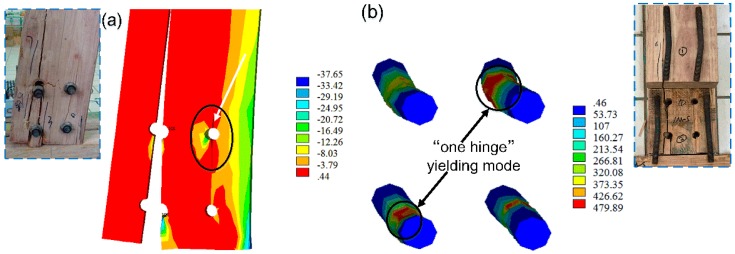
Numerical failure modes: (**a**) cracking of beam component; (**b**) yielding of bolts (all numbers in the figure are in MPa).

**Figure 15:**
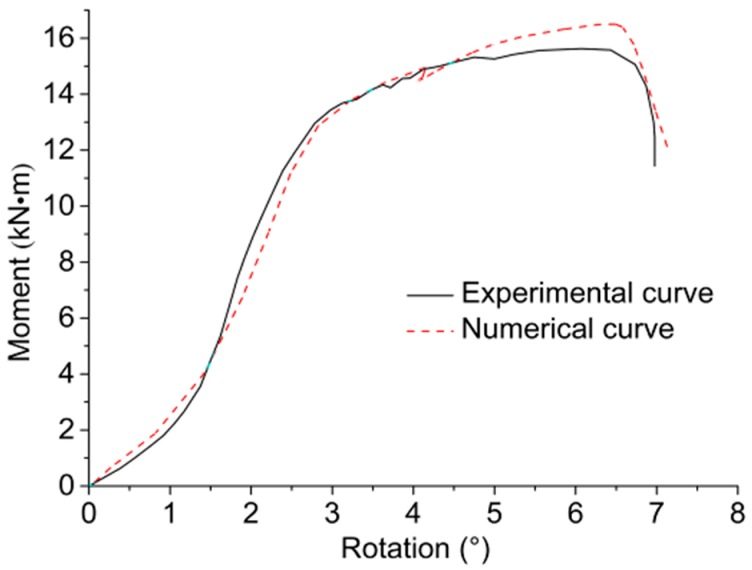
Comparison of experimental and numerical *M-θ* curves.

Figure 13 shows the 3D FEM established by the software package ANSYS. The numerical failure modes are given in Figure 14 with the comparative experimental phenomena. Similar to the experimental phenomena of monotonic tests, varying degrees of crack development were observed on the tension and compression sides with embedment deformation of bolt holes. The stress nephogram shows that the stress at mid-span of bolts had reached the yielding strength when scrimber joints failed, leading to a “one hinge” yielding mode of the bolts. The comparison of numerical and experimental *M-θ* curves is shown in Figure 15, and the results of comparing rotational performance are given in Table 3. It is suggested that the numerical and experimental results are in good agreement with relative errors less than 10%, thus the developed 3D FEM can be an effective tool for further investigations.

### 3.3. Stress Distribution

**Figure 16:**
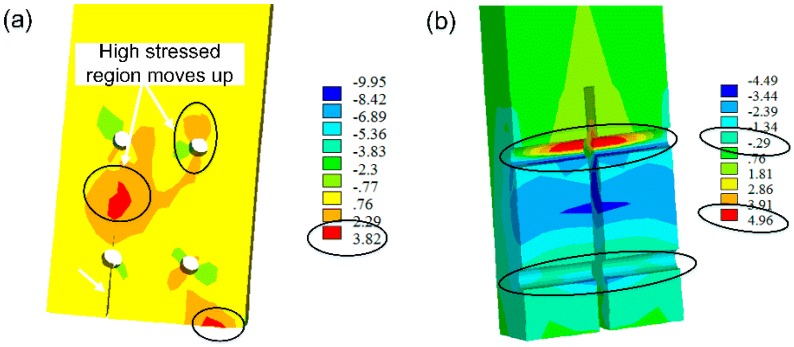
Stress distribution when initial splitting appears: (**a**) perpendicular-to-grain stress; (**b**) shear stress (all numbers in the figure are in MPa).

Figure 16a,b show the perpendicular-to-grain stress distribution and shear stress distribution when initial splitting appears, respectively. It is noted from Figure 16a that high-stressed regions are observed along the row of bolts on the tension side and near the contact segment between scrimber beam and column, where the initial cracks would probably appear according to experimental phenomenon. With the development of cracks, the region of high perpendicular-to-grain stress moves up on the tension side and gradually expands to the upper bolt holes on both sides. Moreover, the maximal perpendicular-to-grain tensile stress (i.e., 3.8 MPa) is higher than the perpendicular-to-grain tensile strength (i.e., 3.4 MPa). Figure 16b shows that the shear stress is symmetrically distributed on both sides of the steel connection plate. However, the maximal shear stress around bolt holes on the tension side (i.e., 4.96 MPa) is only 58% of the shear strength (i.e., 8.6 MPa). It can be concluded that the combination of perpendicular-to-grain tensile stress and shear stress governs the failure mode of bolted scrimber joint.

### 3.4. Parametric Analyses

**Figure 17:**
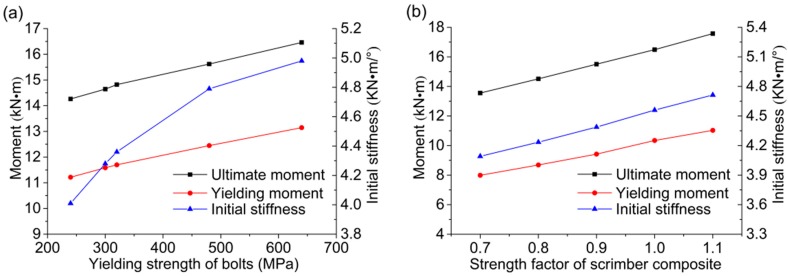
Parametric analyses: (**a**) various yielding strength of bolts; (**b**) various strength factor of scrimber composite.

In this section, parametric analyses were conducted to investigate the influence of two different parameters. The scrimber joints with five different bolts with grades ranging from 4.6 to 8.8 have been modeled, and the rotational performance indexes with various yielding strength of bolts are shown in Figure 17a. The moment resisting capacity is proportionally increased with the increase of bolts grade. However, the growth tendency in initial stiffness becomes slower with the increase of bolts grade. It is suggested that the bolts in grade of 6.8 and 8.8, with the yielding strength of 480 MPa and 640 MPa respectively, are the proper choice in consideration of performance.

The mechanical properties of the scrimber composite can be modified through the impregnation and molding process. To investigate the influence of mechanical properties of the scrimber composite on the joint behavior, all the strength indexes of the scrimber used in this study were multiplied by a factor in a reasonable and achievable range from 0.7 to 1.1. Thus, the strength modification factor is considered as another influential parameter. As shown in Figure 17b, the ultimate moment, yielding moment, and initial stiffness are all proportionally increased with the increase of scrimber strength. Compared to the bolt grade, improving the properties of wood is a more economical and effective way for enhancing the rotational performance of the bolted timber joints.

### 3.5. Comparison between Glulam and Scrimber Joints with the Same Geometrical Dimensions

In this section, the numerical results of scrimber joints and the experimental results of glulam joints with the same geometrical dimensions were compared. The scrimber joint with the beam section of 300 mm × 200 mm and the column section of 300 mm × 250 mm was analyzed. The initial stiffness, ultimate moment, and failure rotation of the scrimber joint were 38.8%, 63.4% and 8.5% larger than those of glulam joint, respectively. Moreover, scrimber joints still perform well in ductility with the ductility ratio equal to 2.16. It is noted that the increase of cross sectional size of the scrimber joints can produce far larger increase of stiffness and load carrying capacity with a smaller decrease of ductility.

## 4. Discussion

The development of scrimber composite can not only increase the value of fast-growing wood, which is widely available in China, but also provide an alternative material for wood-based constructions. A series of experiments were conducted to evaluate the practical applications of such material, and a 3D model was then developed to investigate the stress distribution and failure mechanism. It was noted that the bolted joints built with the scrimber composite performed much better than commonly used glulam joints in initial stiffness, ductility, and energy dissipation. However, when subjected to lateral load, the crack development of scrimber joints was similar to that of glulam joints. Previous researches have shown that the failure of glulam joints is governed by the perpendicular-to-grain tensile stress, which was verified as the key factor for failure of scrimber joints through numerical analysis. These findings suggest that the bolted joints built with scrimber composite have certain similar attributes to bolted glulam joints, which contributed to developing the design procedure of scrimber composite with appropriate reliability levels. Due to the novel manufacturing process, scrimber joints also have some unique attributes such as complicated interactions among the curved layers. The numerical model is suitable for further research on other applications of such material such as floor and shear wall.

## 5. Conclusions

A series of monotonic and low frequency cyclic tests were conducted to investigate the rotational performance of bolted joints built with a wood-based scrimber composite material. Due to the initial clearance between bolts and wood components, the moment-resisting capacity of joints was relatively low at the beginning of loading. With local crushing of the beam on the compression side, the moment-resisting capacity of joints significantly increased after the bolts were sufficiently contacted to the wood components. At large rotations, splitting cracks of the beam developed upwards on the tension side, and the joints almost accessed a plastic deformation phase with the yielding of bolts. The joints eventually failed when the run-through crack appeared along the beam with obvious embedment deformation of bolt holes. Ductile failure with yielding of bolts was observed as the main failure mode for the specimens. These results are due to the production process of the wood scrimber composite bring a considerable increase in its mechanical property (i.e., strength and stiffness), and bolt yielding is therefore obtained as the main failure model and cracking of wood is delayed at the same time. Ductile failure with bolts yielding is therefore obtained as the main failure model of the joints. For the results of cyclic tests, the maximum secant stiffness and energy dissipation capacity were 30% and 90% larger than those of glulam joints, respectively. Considering the dimension of scrimber joints is eventually smaller than that of glulam joints, scrimber joints performed better in both ductility and energy dissipation capacity, which makes the scrimber composite a promising material for timber structures.

A 3D numerical model was then developed and validated by comparisons of *M-θ* curves and failure modes. The proposed FEM was able to capture the bending deformation of bolts and the deformation of wood embedment near the bolt holes. The development of cracks in the high-stress region is also captured by the FEM. Through analysis of different stress distribution combined with crack development, it is suggested the tensile stress perpendicular-to-grain governs the failure mode of the scrimber joints. Results suggested that the initial stiffness, ultimate moment resisting capacity, and failure rotation of the scrimber joints were 38.8%, 63.4%, and 8.5% larger than those of glulam joints with the same member sizes, which further showed the superiority of such scrimber composite material. The presented experimental results and validated numerical model can serve as a fundamental basis and useful tool for evaluating the mechanical behavior of scrimber joints with different configurations in future research, thus supporting more applications of such composite material in structural engineering.

## Figures and Tables

**Table 1:** Comparison of glulam joints and scrimber joints.

Joint Type	Initial Stiffness	Ultimate Moment	Failure Rotation	Ductility Ratio
	*K_α_*	*M_peak_* (KN∙m)	*θ_peak_* (°)	*μ*
Glulam joint [17]	4.49	17.94	6.71	– *
Scrimber joint	4.79	15.62	6.97	2.28

* The ductility ratio of glulam joints is not considered due to its brittle failure mode.

**Table 2:** Mechanical properties of scrimber composite used in the model.

Modulus of Elasticity (N/mm^2^)	Modulus of Rigidity (N/mm^2^)	Strengths (N/mm^2^)	Poisson Ratio –	Fracture Energies (N/mm^2^)
*E_L_* = 16,800 *E_R_* = 1300	*G_LR_* = 1120	*f_t,90_* = 3.4 *f_c,0_* = 84.0 *f_c,90_* = 16.0 *f_v_* = 6.3	*μ_LR_* = 0.028 *μ_RT_* = 0.050 *μ_LT_* = 0.028	$G_{c}^{I}$ = 0.56 $G_{c}^{\mathrm{II}}$ = 1.32

**Table 3:** Comparison of numerical and experimental results.

Analysis Type	Initial Stiffness	Ultimate Moment	Failure Rotation	Ductile Coefficient
	*K_α_*	*M_peak_* (KN∙m)	*θ_peak_* (°)	*μ*
Experimental result	4.79	15.62	6.97	2.28
Numerical value	4.56	16.49	7.12	2.12
Relative error	−4.8%	5.6%	2.2%	−7.1%

## References

[1] Taheri F., Nagaraj M., Khosravi P. (2009). Buckling response of glue-laminated columns reinforced with fiber-reinforced plastic sheets. Compos. Struct..

[2] O’Loinsigh C., Oudjene M., Shotton E., Pizzi A., Fanning P. (2012). Mechanical behaviour and 3d stress analysis of multi-layered wooden beams made with welded-through wood dowels. Compos. Struct..

[3] Quiroga A., Marzocchi V., Rintoul I. (2016). Influence of wood treatments on mechanical properties of wood–cement composites and of populus euroamericana wood fibers. Compos. Part B Eng..

[4] Cabrero J.M., Heiduschke A., Haller P. (2010). Analytical assessment of the load-carrying capacity of axially loaded wooden reinforced tubes. Compos. Struct..

[5] Borri A., Corradi M., Speranzini E. (2013). Reinforcement of wood with natural fibers. Compos. Part B Eng..

[6] Oudjene M., Meghlat E.M., Ait-Aider H., Batoz J.L. (2013). Non-linear finite element modelling of the structural behaviour of screwed timber-to-concrete composite connections. Compos. Struct..

[7] Candan Z., Akbulut T. (2014). Nano-engineered plywood panels: Performance properties. Compos. Part B Eng..

[8] Khelifa M., Celzard A. (2014). Numerical analysis of flexural strengthening of timber beams reinforced with CFRP strips. Compos. Struct..

[9] Tran V. D., Oudjene M., Méausoone P.J. (2015). Experimental and numerical analyses of the structural response of adhesively reconstituted beech timber beams. Compos. Struct..

[10] Jin M., Hu Y., Wang B. (2015). Compressive and bending behaviours of wood-based two-dimensional lattice truss core sandwich structures. Compos. Struct..

[11] Bru D., Baeza F.J., Varona F.B., García Barba J., Ivorra S. (2016). Static and dynamic properties of retrofitted timber beams using glass fiber reinforced polymers. Mater. Struct..

[12] Alaee S.A.M., Sullivan T., Rogers C.A., Nascimbene R. Semi-empirical method to predict the displacement capacity and resistance of cold formed steel frame wood-panel shear walls. Proceedings of the 12th World Conference on Timber Engineering.

[13] Sebastian W., Piazza M., Tomasi R. (2016). State-of-the-art in timber materials and structures research. Constr. Build. Mater..

[14] Feroldi F., Russo S. Structural Behavior of All-FRP Beam-Column Plate-Bolted Joints. J. Compos. Constr..

[15] Li Z., He M., Tao D., Li M. (2016). Experimental buckling performance of scrimber composite columns under axial compression. Compos. Part B Eng..

[16] (2008). Notations for Designations of Iron and Steel.

[17] (2006). Specifications of High Strength Bolts with Large Hexagon Head, Large Hexagon Nuts, Plain Washers for Steel Structures.

[18] (2012). Standard Test Methods for mechanical Fasteners in Wood.

[19] (2012). Standard Test Methods for Cyclic (Reversed) Load Test for Shear Resistance of Vertical Elements of the Lateral Force Resisting Systems for Buildings.

[20] He M.J., Liu H.F. (2015). Comparison of glulam post-to-beam connections reinforced by two different dowel-type fasteners. Constr. Build. Mater..

[21] Muñoz W., Mohammad M., Salenikovich A., Quenneville P. Determination of yield point and ductility of timber assemblies: In search for a harmonised approach. Proceedings of the 10th World Conference on Timber Engineering.

[22] Chui Y.H., Li Y. (2005). Modeling timber moment connection under reversed cyclic loading. J. Struct. Eng..

[23] Bouchaïr A., Racher P., Bocquet J.F. (2007). Analysis of dowelled timber to timber moment-resisting joints. Mater. Struct..

[24] Hong J.P., Barrett J.D. Wood material parameters of numerical model for bolted connections-compression properties and embedment properties. Proceedings of the 10th World Conference on Timber Engineering.

[25] Hong J.P., Barrett J.D., Lam F. (2011). Three-dimensional finite element analysis of the japanese traditional post-and-beam connection. J. Wood Sci..

[26] Audebert M., Dhima D., Taazount M., Bouchaïr A. (2011). Numerical investigations on the thermo-mechanical behavior of steel-to-timber joints exposed to fire. Eng. Struct..

[27] ANSYS, Inc (2013). Release 15.0 Documentation for ANSYS: ANSYS Mechanical APDL Theory Reference.

[28] Franke B., Quenneville P. (2011). Numerical modeling of the failure behavior of dowel connections in wood. J. Eng. Mech..

[29] Alfano G., Crisfield M.A. (2001). Finite element interface models for the delamination analysis of laminated composites mechanical and computational issues. Int. J. Numer. Methods Eng..

[30] Sjödin J., Serrano E., Enquist B. (2008). An experimental and numerical study of the effect of friction in single dowel joints. Holz als Roh-und Werkstoff.

[31] Belytschko T., Wong B.L., Chiang H-Y. (1992). Advances in one-point quadrature shell elements. Comput. Methods Appl. Mech. Eng..

[32] Nascimbene R., Venini P. (2002). A new locking-free equilibrium mixed element for plane elasticity with continuous displacement interpolation. Comput. Methods Appl. Mech. Eng..

[33] Nascimbene R. (2014). Towards Non–Standard Numerical Modeling of Thin-Shell Structures: Geometrically Linear Formulation. Int. J. Comput. Methods Eng. Sci. Mech..

